# Estradiol Signaling at the Heart of Folliculogenesis: Its Potential Deregulation in Human Ovarian Pathologies

**DOI:** 10.3390/ijms23010512

**Published:** 2022-01-03

**Authors:** Stéphanie Chauvin, Joëlle Cohen-Tannoudji, Céline J. Guigon

**Affiliations:** BFA, UMR 8251, CNRS, ERL U1133, Inserm, Université de Paris, F-75013 Paris, France; joelle.cohen-tannoudji@u-paris.fr (J.C.-T.); celine.guigon@univ-paris-diderot.fr (C.J.G.)

**Keywords:** estradiol, estrogen receptors isoforms, granulosa cells, folliculogenesis, polycystic ovary syndrome, granulosa cell tumors

## Abstract

Estradiol (E2) is a major hormone controlling women fertility, in particular folliculogenesis. This steroid, which is locally produced by granulosa cells (GC) within ovarian follicles, controls the development and selection of dominant preovulatory follicles. E2 effects rely on a complex set of nuclear and extra-nuclear signal transduction pathways principally triggered by its nuclear receptors, ERα and ERβ. These transcription factors are differentially expressed within follicles, with ERβ being the predominant ER in GC. Several ERβ splice isoforms have been identified and display specific structural features, which greatly complicates the nature of ERβ-mediated E2 signaling. This review aims at providing a concise overview of the main actions of E2 during follicular growth, maturation, and selection in human. It also describes the current understanding of the various roles of ERβ splice isoforms, especially their influence on cell fate. We finally discuss how E2 signaling deregulation could participate in two ovarian pathogeneses characterized by either a follicular arrest, as in polycystic ovary syndrome, or an excess of GC survival and proliferation, leading to granulosa cell tumors. This review emphasizes the need for further research to better understand the molecular basis of E2 signaling throughout folliculogenesis and to improve the efficiency of ovarian-related disease therapies.

## 1. Introduction

Estradiol (E2) is a steroid hormone that regulates important events occurring during the normal menstrual cycle in women, especially the sequence of ovarian follicle growth and maturation. During the different waves of follicular development, one antral follicle is selected for maturation until ovulation, while subordinate follicles inevitably enter atresia [1,2]. E2 is specifically secreted by the granulosa cells (GC) of developing antral follicles upon follicle-stimulating hormone (FSH) stimulation to strengthen follicular growth and maturation. The increased concentration of E2 contained in the follicular fluid of larger follicles further participates in the selection of a dominant preovulatory follicle [3] and finally triggers the luteinizing hormone (LH) surge required for ovulation [1]. As a result of these major intra-ovarian activities, E2 is considered as an important marker of follicle quality.

E2 mediates its effects principally through the activation of ERα and ERβ receptors that are members of the superfamily of ligand-activated transcription factors [4]. Both receptors act mainly in the nucleus to regulate gene transcription through direct (on putative estrogen response element sequences, ERE) or indirect (though tethering to other transcription factors) DNA interactions. This genomic signaling is further augmented by the non-genomic GPER1 (also known as GPR30 or GPER), which is a membrane-bound G-protein-coupled receptor capable of mediating both rapid and transcriptional events in response to high levels of E2 [5].

Based on the well-established importance of E2 in follicular development and maturation, the current review aims at highlighting the potential role of E2 signaling in two human ovarian pathologies that still need better treatment strategies, such as polycystic ovary syndrome (PCOS) and granulosa cell tumors (GCT). This review gives a special emphasis to the recent advances in the biology of ERβ signaling and to the possible contribution of its different isoforms in those pathologies.

## 2. Estrogen Signaling Is Critical for Human Follicle Quality

Follicle quality relies on the proper growth and maturation of GC that implicate the action of endocrine factors such as gonadotropins and E2. Although the prominent role of E2 in folliculogenesis is now established, its downstream signaling is still unsolved, principally because of a lack of information on the expression of its different receptors (at either mRNA or proteins levels) in human GC of different follicle sizes. Yet, a few immunohistochemical studies in human and non-human primate ovaries reveal a differential expression of ERα and ERβ within the GC of developing follicles; in pre-antral follicles, ERβ but not ERα is detectable, while in mature follicles, both receptors are expressed [6,7]. Then, ERβ would arise as the main mediator of E2 actions in GC.

### 2.1. Human Estrogen Receptors Exist in Multiple Isoforms

ERα and ERβ are encoded by two distinct genes (*ESR1* and *ESR2*, respectively), and the mature transcripts contain eight exons. Similarly to other receptors belonging to the nuclear receptor superfamily, these two receptors are composed of a variable N-terminal transactivation domain (that of ERβ is shorter than that of ERα), a highly conserved DNA-binding domain (DBD), and a C-terminal ligand-binding domain. Many alternatively spliced transcripts for ERα and ERβ have been identified, but a few have been shown to translate to functional protein products [4,8]. ERα exists in three isoforms: a full-length 66 kDa isoform (ERα66) containing 595 amino acids, and two truncated isoforms of 46 (ERα46) and 36 kDa (ERα36) with 421 and 279 amino acids, respectively. ERα46 lacks the transactivation domain AF-1 located in the N-terminal region, while ERα36 lacks both transactivation domains AF-1 and AF-2 (within the C-terminal domain) but retains the DBD and a partial ligand-binding domain (presence of an extra 27-amino acid sequence in its C-terminal region) [9]. ERα46 arises from alternative splicing of the ESR1 gene from exon 2 [10], alternative translation initiation [11,12], or by proteolysis of the native ERα66 [13]. ERα46 and ERα36 both inhibit the transcriptional activity of ERα66 [9,10]. These shorter ERα isoforms mediate rapid estrogen signaling and inhibit the genomic activity of ERα66 by competing with DNA binding sites [9] or by reducing ERα66 AF-1 activity [14]. Although ERα66 expression has been widely demonstrated in the human ovary, that of ERα46 and ERα36 remains unknown.

**Figure 1:**
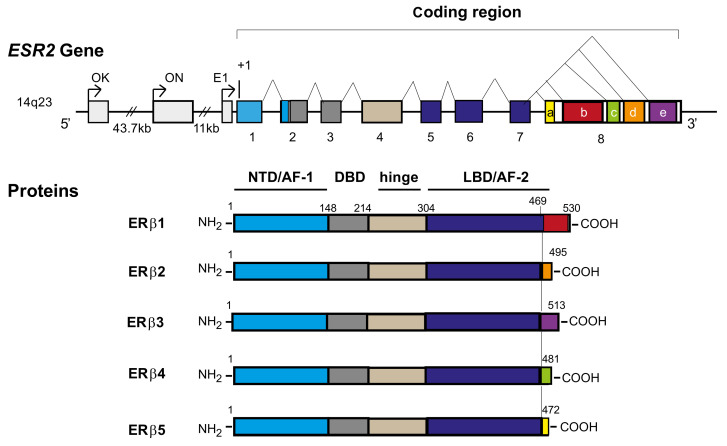
Schematic representation of *ESR2* gene and human ERβ protein isoforms (ERβ1–5). Within the 5′ untranslated region of *ESR2*, three distinct promoters are found (OK, ON, and E1). Exons 1–8 are represented by boxes, and the introns are represented by lines. Exons are represented in different colors, and their encoding protein domains are represented by the corresponding colors. The use of alternative acceptor sites leads to the production of five different proteins (ERβ1, ERβ2, ERβ3, ERβ4, and ERβ5). All these isoforms share common exons 1–7 but differ in their alternative exon 8 (b, d, e, c, and a respectively). For protein isoforms, from the N-terminus to C-terminus, the N-terminal domain (NTD)/activation function 1 (AF-1) is colored in blue, the DNA binding domain (DBD) is colored in dark gray, the hinge domain is colored in clear brown, and the ligand-binding domain (LBD)/activation function 2 (AF-2) is colored in dark blue. At the end of the protein, the alternative splicing produces proteins identical in their first 468 amino acids but differing in the sequence corresponding to the end of the ligand binding of ERβ1, which modifies their capacity to bind ligands. Numbers indicate the amino acids of the protein.

Unlike rodent ERβ spliced isoforms, which include insertions or deletions throughout the middle region of the proteins, human ERβ spliced isoforms (ERβ1, ERβ2, ERβ3, ERβ4, and ERβ5) arise from the specific alternative splicing of exon 8, thereby resulting in sequence variations or truncations at the C-terminal region of the protein [15] (Figure 1). All these isoforms are differently expressed in multiple human tissues as well as in a variety of human cell lines, except for ERβ3, which is selectively expressed in the human testis [16,17]. Alternative splicing for both *ESR1* and *ESR2* genes is species-specific, limiting the relevance of rodent models in the study of the impact of variant isoforms on tissue responsiveness to E2. Indeed, the ERβ2 isoform described in rodents contains in-frame insertions of unique peptide sequence within the ligand-binding domain, resulting in reduced but still detectable ligand-binding capacity [18]. By contrast, alternative splicing in the C-terminal of *ESR2* in humans and primates [19] leads to ERβ isoforms unable to bind E2 [20]. Different studies show that ERβ2, ERβ4, and ERβ5 spliced isoforms cannot form homodimers and bind ligand because of the missing coactivator recruiting helix 12 [20,21,22]. Therefore, the longest ERβ1 isoform is the unique fully active ERβ that binds E2. However, the binding affinity of ERβ1 to E2 is approximately half that of ERα [23], and ERβ1 homodimers show less potent activity on the consensus ERE than ERα homodimers [24]. ERβ2, ERβ4, and ERβ5 have no ligand-dependent transcriptional activity but can heterodimerize with ERβ1 and enhance its E2-mediated transcriptional activity [20]. Their propensity to dimerize in vitro follows the descending order of ERβ1-β4 ≥ β1-β5 > β1-β1 > β1-β2 [20]. In addition, all ERβ isoforms inhibit the transcriptional activity of ERα on an ERE-containing promoter but with various efficiencies (ERβ1 > ERβ2 > ERβ5) [22]. Strikingly, ERβ2 preferentially forms a heterodimer with ERα rather than with ERβ [25] and elicits a dominant negative effect on ERα-mediated transactivation [25] by targeting the heterodimers to the proteasome [26]. ERβ1, ERβ2, ERβ4, and ERβ5 can also promote ligand-independent activity, which is consistent with their intact N-terminal transactivation regions [21,27].

In primate and human ovaries, ERα is diffusely expressed in thecal, interstitial, and in the GC of antral follicles, while ERβ is predominantly located in GC. ERβ expression begins in the GC of immature follicles and reaches maximum levels in maturing and preovulatory follicles [6,28]. Analyses of ERβ isoform mRNA in human also show that all ERβ isoforms are expressed in the ovary [29], in GC [6,30], as well as in various forms of ovarian cancers [31,32], including GC tumors [33]. In addition, ERβ1 and ERβ2 mRNA are differently expressed across the luteal phase with a maximal expression observed in the mid-luteal phase for ERβ1 and in the early luteal phase for ERβ2 [34].

The precise molecular mechanisms regulating ERβ alternative splicing are largely unknown. Still, E2 is described to specifically up-regulate the expression of ERβ4 and ERβ5 mRNA in human GC of antral follicles [30]. ERβ originates from multiple putative promoters (5′-untranslated first exons 0K, 0N, and E1) [35,36] with the same final transcript (Figure 1). mRNAs for each ERβ isoform have different and cell-type specific proportions of the three 5′-untranslated regions (5′-UTRs) that determine their translational efficiencies [37]. These 5′-UTRs may be affected by methylation on GC-rich regions within the promoter [38,39]. Interestingly, the various ERβ 5′-UTRs determine a differential and cell-specific translation inhibition of ERβ expression (0K promoter activation generally possesses a greater inhibitory effect [36]) through various mechanisms such as ribosome stalling or increased mRNA instability [40]. In addition, Smith et al. describe a crosstalk between multiple 5′- and 3′-UTRs in the differential regulation of translation of the different ERβ isoforms [36].

Collectively, these data underline the high complexity and cell specificity in the regulation of ERβ isoform expression that might play important roles in E2 signaling.

### 2.2. Mechanisms of Action of ER

The specificity of E2 action varies depending on the ER distribution pattern in target tissues that express various sets of co-regulators. Several reviews have extensively described how ER can act both in the nucleus and in the cytosol, in the presence or in the absence of E2 [15,41,42,43,44,45,46]. These reviews also provide information on how ER activities can be influenced by several post-translational modifications that can occur in the absence of ligand [47,48,49]. Multiple studies also gave precision on ER genomic activities and showed that homo- or heterodimers involving ERβ and ERα have significantly different target genes [48,50,51,52]. Genomic studies revealed that only one-third of the E2-responsive genes identified so far contain sequences in their promoter that resemble ERE [53,54]. Ligand-bound ER can also regulate gene transcription without directly binding to DNA through protein–protein interactions with other DNA-binding transcription factors such as Jun/Fos (at AP-1 response elements) [55] or SP-1 (at GC-rich SP-1 motifs) or by interaction with the nuclear factor *kappa* B (NFκB) pathway [56]. Strikingly, ERβ possesses a higher ligand-independent activity than ERα, with genomic binding sites containing AP-1-like binding regions associated with ERE-like sites [45,52,54]; ERβ preferentially binds to AP-1 rather than ERE sites. Therefore, in the absence of E2, ERβ would be able to regulate gene expression through non-classical binding sites such as AP-1, while in E2-dominated environment, ERβ may switch over to ERE-based transcription to control the expression of other genes [45]. ERβ1, ERβ2, ERβ4, and ERβ5 also appear to have constitutive transcriptional activity largely independent of E2 binding [4], which adds another layer of complexity in ERβ-mediated signaling.

In addition to its wide genomic effects, E2 is also able to exert non-genomic activities mediated by membrane-associated ER triggering both PI3K and MAPK signaling pathways activation, thereby promoting indirect changes in gene expression [44]. ER proximity to membranes also facilitates interactions with other membrane-associated proteins such as G-proteins, various membrane receptors (e.g., insulin growth factor or epidermal growth factor receptors), or signaling molecules (e.g., Src or PI3 kinase) that initiate crosstalk between ER and other signaling pathways [44].

### 2.3. Role of E2 Signaling in Follicular Growth, Differentiation, and Atresia

Multiple studies demonstrated that during folliculogenesis, ovarian follicles undergo several stages of development and maturation before ovulation. The success of folliculogenesis depends on the action of important regulators, such as locally produced ovarian-secreted growth factors and FSH [57]. Specifically, during antral follicle development, FSH acts on GC to stimulate the expression of the aromatase (*CYP19A1*), which converts thecal-derived C19 androgens to C18 estrogens, principally E2. E2 synergizes with FSH to up-regulate the expression of steroidogenic enzymes and to stimulate GC proliferation and maturation (i.e., by inducing LH receptor (LHR) expression) [58]. Finally, the increased concentration of E2 present in the follicular fluid of large antral follicles participates in the selection of dominant preovulatory follicles and triggers the LH surge necessary to terminate the follicular program and to induce ovulation [58]. Most follicles (>99.9% of follicles) do not reach the ovulatory stage but instead become atretic because of the apoptosis of the GC layer [1,2,58].

Whilst the importance of E2 in follicle development is well established, the role of ER in GC biology remains incompletely understood, particularly in human. However, valuable clues can be provided by transgenic mouse models even if human ERβ isoforms are not conserved in rodents. In mice, the deletion of either ER induces various ovarian phenotypes, from subfertility (*Esr*2^−/−^ mice) due to the disrupted growth and maturation of antral follicles leading to reduced ovulation rate to infertility due to the development of cystic follicles and anovulation (*Esr1*^−/−^ mice); in the case of ERα, this effect on the ovary would be mainly indirect, via its regulation of pituitary gonadotropins, whereas ERβ deletion would impair directly GC proliferation and maturation [59,60,61,62]. In addition, E2 is depicted to prevent GC apoptosis in rodents and cattle [63] and to promote cell cycle progression [64] by regulating Bcl-2, cyclin D, and cyclin E expression [65]. By contrast, in human and primate, E2 possesses intra-follicular atretogenic effects on dominant preovulatory follicles, which can be reversed or not by FSH, depending on the study [3,66]. The observed E2-induced GC apoptosis may be the result of activation of different ER subtypes, especially ERβ isoforms [67,68,69,70]. Indeed, various studies reveal differential effects of ERβ isoforms on cell proliferation and/or apoptosis, such as ERβ2 and ERβ5 that increase prostate or glioma tumor proliferation [68,71], while ERβ1 and ERβ4 induce GC apoptosis [30].

In human, ovulatory defects have been linked to *RsaI* and *AluI* polymorphisms in *ESR2* [72]. These polymorphisms do not result in a change in the amino acid sequence of ERβ protein, but the translation rates or mRNA half-life may be affected [73]. Patients with a loss of function mutation in *ESR2* have been recently described and associated with complete amenorrhea [74,75] or with 46 XY disorders of sex development [76].

All these findings indicate that the differential level of expression of each ERβ isoform may play important roles in E2 action and sensitivity, and they might be involved in various follicular deregulations leading to ovarian pathologies, such as polycystic ovary syndrome (PCOS) and granulosa cell tumor (GCT).

## 3. Importance of E2 Signaling in PCOS

### 3.1. Down-Regulation of E2 Production

PCOS is the most common cause of anovulatory infertility affecting 5–10% of women of reproductive age [77]. In addition to oligomenorrhea/anovulation, women with PCOS are characterized by clinical and/or biochemical hyperandrogenism and polycystic ovaries. PCOS is an endocrine disorder that compromises normal follicular development, with ovaries containing an excess of small antral follicles (≈2–8 mm in diameter) [78]. In these patients, the recruitment and selection of dominant preovulatory follicles are arrested. FSH levels are below the “threshold” level required during the early follicular phase to stimulate normal follicle maturation, resulting in the impairment of follicular maturation. In addition, an increase in LH pulse frequency [79] participates to premature follicle differentiation [80]. Follicular fluid (FF) within follicles of PCOS women exhibit distinct profiles in proteins and hormones than those of fertile women [81,82], and especially a strong reduction of E2 production despite high levels of androstenedione (the natural substrate of aromatase) [83,84,85,86]. The low intra-follicular E2 concentrations [83] may compromise the maturation of developing follicles, thereby favoring the accumulation of a cohort of small antral follicles.

Despite the high prevalence of this disorder, the precise mechanism underlying this phenomenon is poorly understood. Many hypotheses have been formulated, the majority implicating gonadotropin, androgen, and/or growth factor actions. It has been postulated that the elevated circulating LH concentration by itself or the intra-ovarian hyperandrogenism may promote follicle stagnation in the early stages of development by inhibiting the development of a dominant and ovulatory follicle, leading to chronic anovulation and infertility. During physiological follicular development, androgens have a biphasic effect on FSH-induced E2 production in primate GC, i.e., augmentation of FSH action by increasing the expression of *FSHR* [87] in small antral follicles (possibly by up-regulating the expression of ERβ [88]), which is followed by an inhibition of *FSHR* expression in the preovulatory follicle [89]. Studies performed on primary cultures of luteinized GC from large antral follicles of non-PCOS women indeed demonstrate the ability of testosterone to down-regulate *CYP19A1* expression through the androgen receptor (AR) in a dose-dependent manner [90]. Therefore, excess of androgens may directly down-regulate E2 production in PCOS.

Different studies compared the steroidogenic gene expression profile in GC from the follicles of PCOS to those of non-PCOS women undergoing in vitro fertilization. Results frequently diverge depending on the ovarian stimulation protocol used and/or on the number of included patients. In mural GC, *CYP19A1* expression is described to be either unmodified [91,92] or down-regulated [90,93,94] in PCOS when compared to non-PCOS. However, in cumulus GC, *CYP19A1* mRNA levels are higher in PCOS when compared to heathy women [95]. These data suggest the existence of differential *CYP19A1* expression regulations in GC, depending on their proximity to the oocyte. The down-regulation of both *CYP11A1* (encoding cytochrome P450 cholesterol side-chain cleavage enzyme, or P450SCC) and *HSD17B1* (encoding 17β-hydroxysteroid dehydrogenase enzyme, or 17-βHSD) and up-regulation of *SULT1E1* (sulfotransferase Family 1E Member 1) mRNA expression are observed in mural luteinized GC of PCOS as compared to non-PCOS women [93]. P450SCC catalyzes the conversion of cholesterol to pregnenolone, while 17-βHSD and SULT1E1 play a role in estrogen metabolism and biosynthesis [93]. Although we cannot assume that changes in gene expression equate to altered protein expression and function, these data are in favor of a reduction of bioavailable E2 and progesterone in PCOS women.

These comparative studies also measured the expression of gonadotropin receptors and revealed that *FSHR* expression is unmodified [91] or up-regulated [90,96], while *LHCGR* expression is either up- or down-regulated [90,91] in PCOS. Despite these contradictory results, we can assume that high levels of LH [79] along with premature LHR activity [78,97] may contribute to PCOS.

In conclusion, in addition to abnormal levels of LH and FSH, antral follicles of PCOS women are characterized by low levels of bioavailable E2 and progesterone, which probably result from abnormal expression and/or activities of different steroidogenic enzymes present in GC.

### 3.2. Deregulation of Steroid Hormone Receptors Expression

Interestingly, the expression pattern analysis of GPER1 (at protein and mRNA levels) in cumulus GC recently has shown its higher expression in PCOS patients when compared to healthy women [98]. GPER1 is proposed to participate in the inhibition of oocyte meiosis described in this pathology [98]. Two studies report an increase in both ERα (*ESR1*) and ERβ (*ESR2*) expression in mural GC of PCOS women [93,99], while a third one described the opposite [100]; these discrepancies can stem from the difference in the ovarian stimulation protocol used. These investigations did not quantify the expression levels of each ERβ isoform present in luteinized GC [30]. Therefore, further data on the respective expression level of ERβ isoforms in GC from PCOS and non-PCOS women will be helpful to better understand E2 signaling in PCOS. In addition, it is postulated that epigenetic alterations in the *CYP19A1* gene would modify ERβ binding to the DNA and prevent aromatase expression in PCOS [95]. Therefore, an alteration of ERβ isoforms expression and/or DNA accessibility may participate in this pathology. Meanwhile, deregulations of the expression of other sex steroid hormone receptors are similarly described in PCOS, with either an unmodified [91] or an up-regulation [96] of AR expression and a down-regulation of progesterone receptor A expression [100] in mural GC from PCOS follicles.

Taken together, these different studies demonstrate the existence of various alterations in GC hormonal receptivity within PCOS follicles that could markedly modify GC functions.

### 3.3. Deficiency in Aromatase Activity and Role of Follicular Fluid Components

Studies examining the relationship between aromatase activity and E2 production in PCOS follicles report interesting findings. Indeed, primary cultures of GC from PCOS follicles are responsive to FSH in vitro since they produce markedly increased levels of E2 [78,86]. These cells also show premature LH responsiveness (early acquisition of LHR) leading to progesterone synthesis [97]. These data suggest that endogenous inhibitors may be responsible for the lack of in vivo aromatase function. The composition of FF may have an impact on aromatase activity; FF from dominant follicles (as opposed to non-dominant follicles) has the ability to decrease aromatase activity [101]. FF compounds that negatively affect aromatase activity were investigated and revealed to be principally steroid hormones. Indeed, FF from small (5–8 mm diameter) PCOS or healthy follicles, pretreated with charcoal to eliminate steroids, increase GC aromatase conversion activity, at least partially through increasing substrate affinity (K_d_ of [^11^C] vorozole lower in charcoal-pretreated FF compared to untreated FF). Although charcoal treatment leads to the depletion of additional substances, these findings led to the proposal that steroids could act as endogenous inhibitors of aromatase activity [102]. Among steroids, Agarwal et al. reported that the high concentration of 5α-reduced androgens in FF of PCOS follicles can decrease aromatase activity in primary cultures of GC [86].

In addition to steroids, other factors, which are highly present in the FF of PCOS (when compared to normal matched for FF-volume), can also down-regulate aromatase activity, such as anti-Müllerian hormone (AMH) [103], epidermal growth factor EGF [104], or/and interleukin-6 (IL-6) [83,105]. During follicle development, AMH is produced exclusively by GC, starting at the primary follicle stage, progressively surging up to a peak in small antral follicle and then gradually disappearing in large follicles, except in cumulus GC. AMH inhibits FSH-induced E2 production by GC through inhibition of the catalytic activity of adenylate cyclase [87], therefore preventing the growth and selection of follicles in PCOS [106]. This direct relationship between AMH and E2 can be determined since the serum E2 level starts to rise only once the serum AMH level passes under some threshold [107]. Meanwhile, EGF and IL-6 are also potent inhibitors of E2 and progesterone production in human GC [108,109] and could synergize with AMH to decrease aromatase expression/activity through different molecular mechanisms. Indeed, the rapid effect (within 2 h) of FF to decrease aromatase activity in GC could involve non-genomic mechanisms through aromatase phosphorylation [102]. Actually, a conserved phosphorylation site (serine 118 in human) has a significant effect on decreasing aromatase activity and might be an important process in regulating aromatase activity in GC [110].

Hence, all these data indicate that despite high intra-follicular concentrations of androstenedione and bioactive FSH in PCOS follicles, 5α-reduced androgens together with other factors such as AMH, EGF, and/or IL-6, may decrease aromatase activity within GC to down-regulate E2 production in vivo.

### 3.4. Potential Effect of Low E2 Concentrations on FF Composition

**Figure 2:**
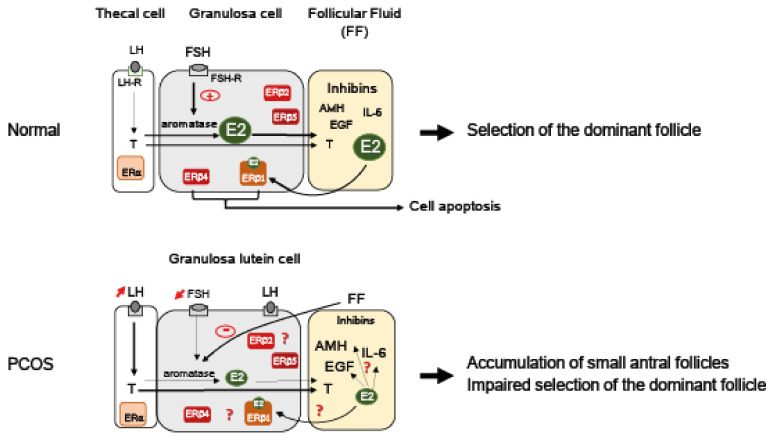
Working model for the role of E2 signaling in PCOS follicles. In granulosa cells (GC) of small antral follicles, FSH stimulates the expression of the aromatase that converts thecal-derived androgens (i.e., testosterone (T) produced upon LH stimulation) to estrogens (E2). E2 contained in the follicular fluid (FF) synergizes with FSH to promote follicular growth and further participates in the selection of the dominant preovulatory follicle [1,3]. The FF of normal antral follicle contains a large set of proteins and hormones such as high levels of inhibins and E2 together with low concentrations of anti-Müllerian hormone (AMH), interleukin-6 (IL-6), epidermal growth factor (EGF), or T. Contrary to thecal cells that principally express ERα, GC mainly express ERβ isoforms [29,30]. Among them, only ERβ1 binds to E2 [20]. ERβ1 as well as ERβ4 possess pro-apoptotic activities that may influence follicles’ fate [30]. In PCOS follicles, abnormal high levels of LH stimulate the T production of thecal cells. In addition to deficit in FSH, endogenous inhibitors (principally steroids) present in the FF [101,102] down-regulate aromatase activity, resulting in lower E2 production. Lower levels of E2 might participate in a higher production of AMH, IL-6, and/or EGF present in PCOS FF [83,105,115,118,119]. Low intra-follicular E2 concentrations may compromise the maturation of developing follicles and favor the accumulation of small antral follicles. The GC of PCOS follicles prematurely express LH receptors (LH-R) [80], which participate in the early maturation of these follicles. The expression levels of each ERβ isoform are still unknown. Specific ERβ isoforms expression might influence follicular fate and contribute to the high number of follicles that characterizes this pathology [78]. LH: Luteinizing hormone; FSH: Follicle-stimulating hormone; LH-R: LH receptor.

In addition to displaying reduced concentration of E2, the FF of PCOS woman follicles are also characterized by a reduction of inhibin A and B concentrations when compared to the FF of sized-matched follicles of healthy women [111]. Inhibin A and inhibin B secretions are regulated differently in vitro, with FSH stimulating inhibin A, but not inhibin B, secretion from human GC primary cultures [112]. Therefore, deficiency in FSH stimulation is probably not the only etiology of both inhibin A/B reduction in PCOS. On the other hand, E2 is an interesting regulator candidate, since it stimulates inhibin B expression [113]. One could speculate that the reduced production of E2 measured in PCOS follicles may contribute to the lower levels of inhibin B measured in the FF. Contrary to inhibin, IL-6 increases significantly in the FF of PCOS follicles [83,105], and this cytokine is known to decrease E2 production by reducing *Cyp19A1* expression in rat GC [114]. Since E2 is able to inhibit NFκ-B-mediated up-expression of *IL-6* [115], its lower production in PCOS could reinforce the higher production of IL-6 observed in FF of PCOS follicles. In addition, E2 is also able to down-regulate TGFβ secretion [116] that usually acts as a potent inhibitor of thecal androgen production in rat [117]. Considering that these regulations might be conserved in human, one could postulate that the decrease in E2 production measured in PCOS could stimulate androgens production in thecal cells. Altogether, bidirectional relationships between E2 concentration and that of growth factors (TGFβ [116], EGF [118]), AMH [119], or IL-6 [115] present in PCOS FF highlight the potential role of E2 signaling in PCOS and complicate the understanding of its etiology (Figure 2).

### 3.5. Potential Role of E2 Reduction on GC Survival

GC survival is described to be altered in PCOS [120,121]. Analysis of GC from small follicles (4–8 mm diameter) of PCOS and non-PCOS patients reveal a decreased expression of the apoptotic effector caspase-3 together with an increased expression of the anti-apoptotic survival factor cIAP-2 (cellular inhibitor of apoptosis protein 2) in PCOS [120]. On the other hand, GC from larger (16–18 mm diameter) PCOS follicles show a higher apoptosis activity (activation of Forkhead box O3) than those of healthy ones [121]. Thus, these data suggest that depending on the size of the follicle, GC are differently sensitive to apoptotic signals, which possibly lead to differential follicle fate in PCOS.

Among the factors that could regulate GC fate in PCOS, one could cite AMH. Indeed, a protective effect of AMH on follicle atresia has been recently described with AMH-target genes being differently regulated in GC of PCOS compared to non-PCOS women [122]. E2 may also contribute to GC fate in PCOS, as depicted in rhesus monkeys, high concentrations of E2 inducing atresia of antral follicles [3]. In addition, E2 specifically regulates the expression of ERβ isoforms, of which two can trigger apoptotic events in GC [30]. E2, by regulating specific ERβ isoform expression in GC of PCOS, might participate in their follicular fate.

Further studies are still needed to better understand whether PCOS follicles are indeed protected from atresia. If it is confirmed, these processes would then contribute to the higher number of follicles that characterizes this pathology.

## 4. Roles of Estrogen in GCT

### 4.1. Most GCT Are E2-Secreting Tumors

GCT are the most common type of potentially malignant ovarian sex cord-stromal tumor that represent 5% of all ovarian malignancies [123]. These tumors are proposed to arise from the default of apoptosis and rapid proliferation of GC from growing follicles destined to atresia [124,125]. There are two distinct subtypes of GCT with different clinical and histopathological parameters: the juvenile (diagnosed around puberty) and the adult (frequently peri- and post-menopausal women) GCT. These tumors are characterized by their slow growth and late recurrence. They are usually treated by surgery alone and have a good prognosis (a 5-year survival rate from 75 to 90%). However, recurrence is associated with poor prognosis, since more than 80% of patients will die. GCT are functional tumors that may produce excessive levels of E2 either because of the up-regulation of *CYP19A1* expression [126,127] or the high number of tumor cells producing E2 [125]. This hyper-estrogenism is responsible for GCT-specific clinical signs such as abnormal vaginal bleeding and precocious puberty [123]. Although E2 is the hormone responsible for the observed clinical manifestations and can be used as a reliable GCT marker, its fluctuating levels in patients cannot predict the tumor activity [128]. GCT show FSH-like growth stimulation despite low FSH levels and the absence of activating mutations in *FSHR* gene [124], with a high production of E2 and inhibins. Excess of inhibins production exerts negative feedback on FSH secretion that becomes unusually low. Serum inhibins as well as AMH levels are frequently abnormally high in patients with GCT [128]; combining AMH levels with inhibins estimation is shown to improve the detection of recurrent disease [129].

The exact etiology of GCT has not been elucidated, and the molecular switches that trigger the progression of the disease in advanced stages remain unresolved. Molecular genetic studies propose that these tumors have a somatic missense point mutation (c.402C > G) in the *FOXL2* (Forkhead box L2) gene, which is observed in 97% of adult GCT and in 5% of juvenile GCT [123], which represents a key element that distinguishes juvenile from adult GCT. FOXL2 is an essential transcription factor controlling the proliferation, apoptosis, and steroidogenesis of GC [130]. FOXL2 mutation (C134W) results in *CYP19A1* up-regulation, probably through ERβ [131], leading to high E2 production [132]. FOXL2 mutation impacts on many signaling pathways involved in GC proliferation and apoptosis, i.e., inducing the suppression of follistatin expression or blockage of caspase-8/Bak (Bcl-2 family member)-related apoptotic pathways [133]. Recently, our study on a GCT mouse model suggests that the disruption of p53/Rb (retinoblastoma protein) signaling could drive tumor initiation and growth in antral follicles [125].

### 4.2. Role of E2 Signaling in GCT

The direct contribution of E2 in GCT oncogenesis is still largely unknown. Since GCT account for about 5% of ovarian malignancies with an incidence of 0.52 to 1.6 per 100,000 women per year [128], studies about GPER1 and steroid hormone receptor distributions in GCT are quite limited. Contradicting patterns of GPER1 expression show that 14% to 90% of GCT express GPER1 [134,135,136] with either an up-regulation [136] or an unchanged expression between primary and recurrent tumors [134,135]. On the other hand, ERβ is reported to be preferentially expressed in GCT [31,136,137] along with a high expression of AR (59% of positive cells are AR positive [138]) and PR (98–100% of positive cells are PR positive), while ERα is present at moderate to high levels (20–66% of GCT are ERα positive) in these tumors [31,136,139,140,141,142]. These different observations may partly be explained by the small patient samples, the use of arbitrary cutoff points for interpreting positive immunoreactivity, and the lack of valuable antibodies [143]. Paired analysis of a small number of primary and recurrent samples from the same patient demonstrate a significantly stronger ERβ immunoreactivity in the recurrent GCT compared to the primary tumor [136]. However, the absence of ERβ in primary GCT is characterized by a worse prognosis [137].

All these reports assessed total ERβ or ERβ1 [136,137,139], except for two studies that show a strong expression of ERβ2 and ERβ5 in GCT [31,33]. ERβ1, ERβ2, and ERβ5 are highly expressed in the nucleus of all studied GCT cells [33]. ERβ2 is also depicted to display a punctate distribution in the cytosol that reflects a mitochondrial localization. Interestingly, ERβ2 is able to interact with proteins of the Bcl-2 family [33], which are highly detected in GCT [144] and play an important role in preventing cell apoptosis. ERα and ERβ are indeed described to be present in the mitochondria of various cell types and tissues [145], being able to regulate mitochondrial activities [146]. Our search in Mitoproteome, a human mitochondrial protein database (www.mitoproteome.org accessed on 23 December 2021), yields no results for ERα but reveals the presence of all isoforms of ERβ (unpublished observations). These data suggest a distinct function between cytoplasmic and nuclear ERβ through genomic, non-genomic, and mitochondrial mechanisms.

Although various estrogen receptors are expressed in GCT, the role of E2 in this form of tumor has been purely speculative for a long time. Based on the idea that E2 promotes tumor growth in various cancers [41], anti-estrogen therapies with aromatase inhibitors are currently used in patients with recurrence [147]. Studies on human metastatic and primary granulosa tumor cells (KGN, COV434) did not support a growth-stimulating effect of E2 [33,135,136,148]. However, in these cells, ER-mediated transactivation is not functional because of the constitutive activation of NFκ-B [135,148]. In this context wherein ER signaling is inactive, we observed that E2 decreases KGN cell migration through GPER1-mediated ERK1/2 inactivation, therefore possibly preventing GCT metastasis spreading [135]. Two studies, including ours in a mouse GCT cell line wherein ER signaling is active, support the idea that E2 favors the growth of GCT by promoting cell survival [136,142]. This E2-mediated effect may be triggered by ERα, acting either alone or in combination with ERβ [142]. The ERα-dependency for the pro-survival action of E2 in GCT may explain the relatively limited clinical benefits of aromatase inhibitors, since not all GCT express ERα [31,136,139,140,141,142]. In addition, this effect of ERβ in GCT would be different from that described in other cancers, wherein this receptor is generally described as an anti-proliferative factor that is down-regulated in breast (BC), prostate (PC), and epithelial ovarian cancers (OC) [67,69,149]. ERβ that commonly corresponds to ERβ1 is generally presented as a tumor suppressor that blocks cell proliferation and induces cell apoptosis [150]. However, the role of ERβ as a tumor suppressor is still controversial and may be tissue-dependent. ERβ may have a bi-faceted role as reported in BC cancers, in which ERβ activation either prevents or promotes cell growth, depending on the presence or the absence of ERα, respectively [69]; when present together with ERα, ERβ generally has a restraining effect on ERα activities [69], contrasting with its action in GCT [142]. Since ER homo- and heterodimers regulate different sets of downstream target genes, the ER expression ratios (ERα: ERβ isoforms) may then be an important determinant in ER-mediated signaling pathways. On the other hand, ERβ effects might be even more complex than they appear when considering each ERβ isoform. Indeed, higher mRNA levels of ERβ2 than the ERβ1 isoform are correlated with better survival in late-onset ERα-positive BC [151], whereas high mRNA levels of ERβ1 and ERβ5 may contribute to poor survival of patients with ERα-negative BC tissue [152]. GCT express different ERβ isoforms [33], which could greatly influence tumor progression. One could speculate that the ligand-insensitive ERβ isoforms could participate in cell growth independently of E2. Further investigations are needed to determine whether ERβ isoforms could play a role in GCT growth. An additional level of complexity of these regulations arises from ERβ subcellular localization that could also account for different effects. Two independent and contradictory studies on OC report that the presence of ERβ in the cytosol is an unfavorable prognostic factor for disease-free survival [153,154], while another depicts that nuclear ERβ shortens progression-free survival [155]. Focusing the analysis on the subcellular localization of ERβ isoforms can also give various results depending on tumor types. Indeed, a large PC sample analysis shows that the majority (>80%) of the specimens are positive for cytoplasmic ERβ1 and nuclear ERβ2 (nERβ2), and 36% are positive for cytoplasmic ERβ5 (cERβ5) [156]. Patients positive for both ERβ2 and ERβ5 (nERβ2+ and cERβ5+) exhibit a worst clinical outcome. The ectopic expression of ERβ2 and ERβ5 in prostatic cell lines further uncovers that these two isoforms are strongly associated with PC metastasis [68,156] and chemotherapy resistance [157]. By contrast, in BC and OC, cytosolic ERβ2 localization correlates with shorter overall survival at 15 years and with chemoresistance [32,158,159]. Discrepancy in correlations between ERβ isoforms expression/localization and function between BC/OC and PC suggests a fundamental difference in the role played by E2 signaling in these pathogeneses. All these studies reveal that there are ER subtype-specific expression changes in cancer that varies depending on tumor type and disease stage. These findings suggest that according to the different pathological circumstances, various binding partners, such as hormone receptors [160] or other proteins [161], could modify ERβ functions and then lead to different outcomes.

### 4.3. ERβ as A New Therapeutic Target?

Hormone therapy has limited success in the treatment of recurrent GCT [147], which can be attributed to the relative expression levels of ERα and ERβ isoforms. The ratio of ERα to ERβ correlates with progressive steps along tumorigenesis. However, to assess whether hormone receptivity is still present or lost during disease progression or chemotherapy, it would be of high interest to undertake a larger analysis of the expression profiles of hormone receptors between primary diagnosed and relapsed cases. These investigations would help to improve patient therapies. PR and AR are frequently highly expressed in GCT and can influence the action of ER [160], and they could explain the observed variation in the responsiveness to anti-estrogen therapies [147]. Further studies aiming at examining whether inhibition of both ER and AR could be efficient in the treatment of GCT need to be evaluated [136]. Strategic coupling of agents targeting several pathways should be considered for a greater efficacy in therapy.

Botanical estrogens are widely consumed in the diet or as dietary supplements by women and preferentially bind to ERβ (lost at high concentrations, >1 µM) [162]. They still bind ER with a 1000-fold lower affinity than E2 [162]. Interestingly, Liu et al. report a significant growth inhibition of natural ERβ agonists (liquiritigenin, Liq, and S-equal) through pro-apoptotic actions on OC [163]. When compared to E2-modulating gene expression in MCF-7 breast cancer cells, ERβ botanical agonists are depicted to be less stimulatory on gene expression promoting proliferation and motility, while they are more effective in up-regulating apoptotic genes [164]. These data open a promising perspective and need to be investigated on GCT growth.

The high expression of ERβ observed in GCT suggests that E2 could also act on a tumor environment [123]. The growth of tumors is known to be dependent on neovascularization to provide the tumor with all required nutrients [165]. Therefore, it would be interesting to explore the role of E2 on angiogenesis. Angiogenesis is an essential process in normal tissue growth, and it involves multiple factors such as vascular endothelial growth factor (VEGF), platelet-derived growth factor β (PDGFβ), or the basic fibroblast growth factor (bFGF). Interestingly, the introduction of ERβ1 into malignant BC injected orthotopically in immunodeficient mice inhibits their growth and prevents tumor expansion by inhibiting angiogenesis through the down-regulation of both PDGFβ and VEGF expression [166].

**Figure 3:**
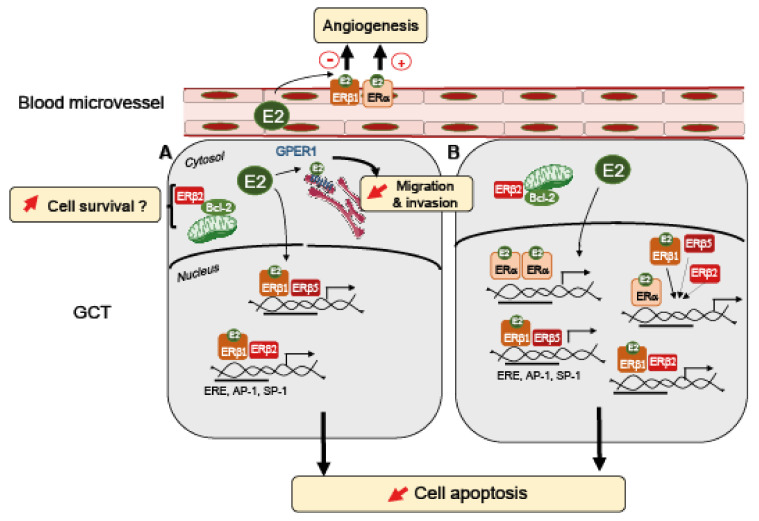
Working hypothesis for a role of E2 signaling in GCT. ERβ is expressed in all GCT [31,136,137]. There is a heterogeneity of ER subtypes expression among GCT [31,136,137,139,140,141,142]. In GCT, all ERβ isoforms are localized in the nucleus [31,33], except for ERβ2, which is also present in the cytosol, possibly interacting with mitochondrial Bcl-2 [33] to promote GCT survival. GCT produce E2 that could bind to GPER1 and promote the inhibition of GCT migration and invasion, in the absence of ERα [135] (**A**). In addition, E2 could bind to ERβ1, which would either homodimerize or heterodimerize with ERβ5 or ERβ2 [20], in the absence of ERα, to regulate pro-survival or pro-apoptotic gene expression, through DNA interactions with ERE, AP-1, or SP-1 sites [56]. ERβ1, ERβ2, and ERβ5 might also trigger non-genomic pathways that still need to be identified in GCT. (**B**) In the presence of ERα, E2 binding would induce its homo- or heterodimerization with other ER subtypes that would finally regulate the expression of other sets of pro-survival genes. ERβ isoforms inhibit the transcriptional activity of ERα [22]. In addition, E2 could also bind to ERβ1 or ERα, which are expressed in endothelial cells, to respectively reduce or stimulate angiogenesis [166,167]. The presence of specific ER subtypes probably orientates the fate of GCT.

In conclusion, all these studies point out the importance of determining the exact hormone receptor status (ERα, ERβ isoforms, GPER1, AR, PR) in GCT that could vary between patients and tumor stages (Figure 3). Improvement in getting this information will ensure the use of relevant hormonal therapies yielding higher success in GCT treatments.

## 5. Concluding Remarks

In this review, we highlight the potential roles of E2 in two human ovarian pathologies. We provide evidence that the ratio of ER expression, especially that of ERβ isoforms, is crucial for GC proliferation and apoptosis and may be essential in the development of normal or tumor-derived follicles. The existence of a highly complex E2 signaling with multiple ERβ isoforms suggests that yet unidentified deregulation could contribute to subtle endocrine-related ovarian disorders causing idiopathic infertility.

One of the important future challenges is to detect each ERβ isoform protein in single-cell analyses, but it requires reliable isoform-specific antibodies that are not yet available. Therefore, alternative antibody-free techniques must be developed to scrutinize ER heterogeneity in situ within different follicles or within GCT. In addition, assuming that ERβ isoforms (especially ERβ2, ERβ4, and ERβ5) possess large genomic activities independently of the presence of E2, new programs of in silico designed drugs need to be carried out, focusing on other conformational situations than the optimal E2-bound ERβ. Hence, the study of the potency of natural agonists or the development of new drugs directly regulating specific ERβ isoform activities needs to be considered. It would lead to the emergence of useful tools to restore GC functions that are deregulated in ovarian-related pathologies as well as in other diseases.

## Data Availability

Not applicable.
